# Leiomyosarcoma of the gallbladder—A case report and a review of literature

**DOI:** 10.1016/j.ijscr.2019.11.062

**Published:** 2019-12-13

**Authors:** Christoph Paasch, Muharrem Salak, Thomas Mairinger, Franz Theissig

**Affiliations:** a Department of General, Visceral and Cancer Surgery, Helios Klinikum Berlin-Buch, Berlin, Germany; b Department of General, Visceral and Minimal Invasive Surgery, DRK Klinikum Mitte, Berlin, Germany; c Institute of Pathology, Helios Klinikum Emil von Behring, Berlin, Germany

**Keywords:** Epitheloid leiomyosarcoma, Sarcoma, Histomorphology, Immunohistochemistry, Histogenesis, Tumor of gallbladder

## Abstract

**Introduction:**

Primary sarcomas of the gallbladder (GB) are a rare disease that were first described by Griffon and Segall in 1897. Leiomyosarcomas (LMS), as described in the case report at hand, are considered a major subgroup.

**Presentation of case:**

A 62-year-old female was referred to our hospital with intermittent right upper quadrant pain. A 45 mm mass arising from the neck of the GB was diagnosed by ultrasound. No distant metastases were revealed. We successfully removed the GB and the surrounding liver tissue. We also performed a lymphadenectomy of the hepatoduodenal ligament. The histopathological and immunohistochemical examination revealed an R0 resected epithelioid LMS of the GB.

**Discussion:**

Our review of literature shows only 20 publications of LMS of the GB. The majority of the patients are female with an average age of 65.95 years. In a non-metastasized stage, a cholecystectomy with a wedge resection of the surrounding liver tissue, accompanied by a lymphadenectomy of the hepatoduodenal ligament, is described as a successful surgical approach.

**Conclusion:**

The LMS should be taken under consideration when diagnosing a tumor of the GB.

## Highlights

• A leiomyosarcoma may appear in the gallbladder.
• Leiomoysarcomas (LMS) are considered as a major subgroup of sarcomas.
• In a non-metastatic stage, the cholecystectomy with wedge resection as well as lymphadenectomy of the hepatoduodenal ligament may be sufficient surgical approach.

## 1 Introduction

Primary sarcomas of the gallbladder (GB) are a rare disease that were first described by Griffon and Segall in 1897 [1]. Women are more often affected than men. In the majority of published cases, primary sarcomas of the GB appear between the 6th and 7th decade of life [2,3]. Leiomyosarcomas (LMS), as described in the case report at hand, are considered a major subgroup [[2], [3], [4]].

The work has been reported in accordance with the SCARE criteria [5].

## 2 Presentation of case

**Fig. 1:**
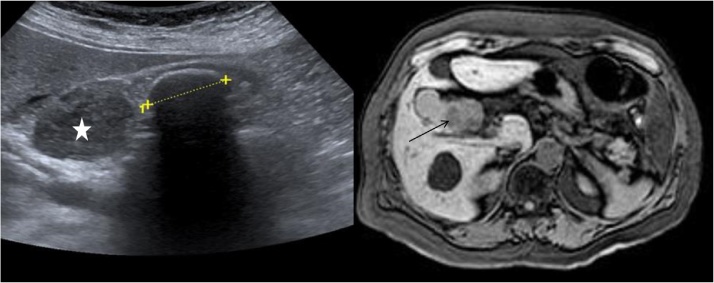
A 4,5 cm mass of the neck of the GB can be seen (marked with a star) on the left side. A gallstone is located next to the tumor. The MRI reveals a tumor marked by the black arrow.

**Fig. 2:**
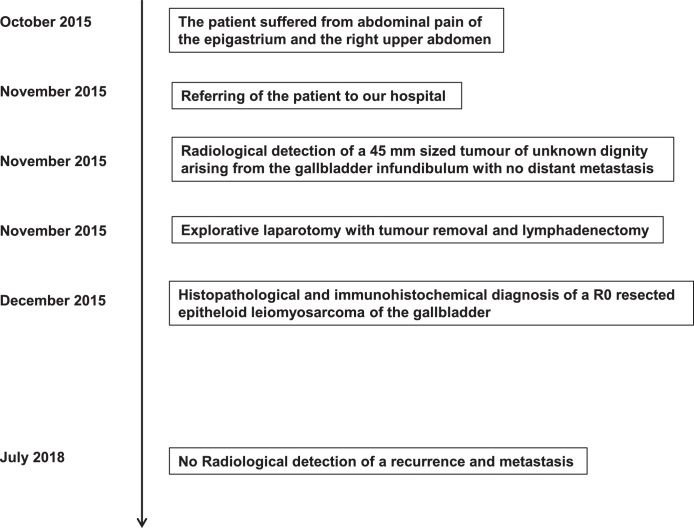
Case report timeline.

A 62-year-old female was referred to our hospital due to intermittent right upper quadrant and epigastric pain for 7 days in 2015. Her medical history consisted of hypothyroidism and pyelonephritis. Her surgical history included an appendectomy. During the clinical examination a positive murphy sign was found. The laboratory tests demonstrated an elevated bilirubin, y-glutamyl transferase, alkaline phosphatase, and lipase. Additionally, the inflammatory markers were elevated. The patient underwent an abdominal ultrasound. An inflamed GB with multiple gallstones was detected. Moreover, a 45 mm mass arising from the neck of GB was found (Fig. 1). Therefore, we conducted an upper endoscopy. No choledocholithiasis was detected. The 45 mm sized tumours had a space-occupying effect on the stomach. The endosonography, MRI and abdominal CT scan did not show distant metastases (Fig. 1, Fig. 2). The mass of unknown dignity showed a contrast agent uptake, but no signs of an infiltrative growth pattern. After a multidisciplinary discussion within our tumor board, the decision was made to remove the mass, with the suspicion of finding a malignant entity. Intraoperatively, neither liver metastases nor the infiltration of the omental fat were revealed. We resected the inflamed gallbladder, as well as the fossa. Additionally, we performed a lymphadenectomy of the hepatoduodenal ligament. The frozen section analysis of the excision margins of the cystic duct did not show any malignant cells.

### 2.1 Pathological findings

**Fig. 3:**
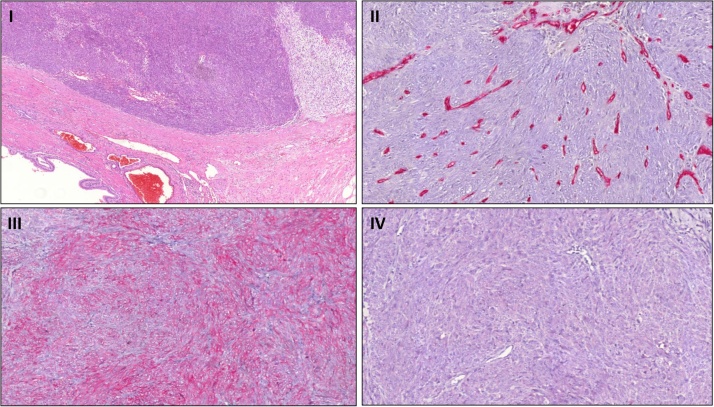
Immunohistochemical detection of DOG1, Calponin and Caldesmon.

The histopathological examination diagnosed an R0 resected 40 mm smooth bounded spindle-shaped epithelioid tumor in a chronic inflamed GB. The liver tissue and lymph nodes did not contain malignant cells (Fig. 3, Picture I).

### 2.2 Immunohistochemical findings

The immunohistochemical examination revealed the expression of DOG1, Calponin and Caldesmon (Fig. 3, Picture II–III).

The postoperative course was uneventful. We discharged our patient 12 days after surgery.

As a follow-up approach, we decided to perform a CT scan every 6 months. No tumor recurrence or metastases were detected up to this day. Our patient remains alive and in good health.

## 3 Discussion

Primary sarcomas of the GB are a rare malignancy. The majority of these sarcomas are leiomyosarcomas. An incidence is estimated as 1.4 per 1000 malignancies of the GB [6,7].

To reveal further knowledge on epidemiology, therapy and outcome of patients with LMS of the GB, we reviewed the literature using the search terms “Sarcoma,” “Leiomyosarcoma,” and “Gallbladder”, with Google Scholar and PubMed. The search yielded 30 relevant publications [1,2,4,[6], [7], [8], [9], [10], [11], [12], [13], [14], [15], [16], [17], [18], [19], [20], [21], [22], [23], [24], [25], [26]]. We excluded publications without available abstracts or insufficient information regarding the patient’s medical history. 10 publications were excluded (Table 1). The articles’ publication dates range from 1982 to 2018. Among the 20 publications, the medical history of 24 patients who suffered from a LMS of the GB was reported. In 23 cases, the LMS occurred primarily in the GB. 18 patients were female, and 6 patients were male with an average age of 65.95 years. The diameter of the mass ranged between 2 and 8 cm. Egorov et al. published a case of a large LMS that weighed 1500 g [22]. In 4 cases, the LMS infiltrated the surrounding organs.

**Table 1:** Publications on Leiomyosarcoma (LMS) of the gallbladder.

Author	Year of publication	Gender	Age	Location	Size *cm*	Pathological findings	Treatment	Outcome
			*Years*			*Secondary/Primary LMS*		*month of survival after surgery*
Guo et al.	2016	female	41	NR	5 × 4,2 × 2,5	Secondary	CCE	>30
Savlania et al.	2012	female	50	Fundus and Corpus	5 × 4 × 3	Primary	CCE, Resection of liver segment IVb + V	12
Im et al.	2014	male	82	Corpus	5.5	Primary	CCE, hepatic wedge resection via laparotomy	NR
Park et al.	2012	male	54	NR	7 × 5,5	Primary	CCE, Resection of liver segment IVb + V, omentectomy, small bowel segmental resection, lymphadenectomy *ligamentum hepatoduodenale*	3
Sherine et al.	2010	female	50	NR	NR	Primary	CCE	NR
Al-Daraji et al.	2009	female	36	NR	3,0	Primary	NR	20
		female	81	NR	4.4	Primary	NR	5
Garcia et al.	2009	male	79	NR	NR	Primary	CT-guided percutaneous drainage due to abscess and cholecystitis	< 1, DOD
		female	81	NR	NR	Primary	Explorative Laparotomie with gastrojejunostomy and ileotransversostomy and biopsy of GB	< 1, DOD
Husain et al.	2009	female	82	NR	4	Primary	Chemotherapy	NR
Ieda et al.	2006	female	78	Invasion of liver, duodenum	NR	sPrimary	CCE, distal gastrectomy, hepatectomy of the IVa, V, partial resection of transverse colon and duodenum	NR
Perez-Montiel et al.	2004	female	32	Fundus	2 × 2 × 1,5	Primary	CCE	> 24
Katagiri et al.	2003	male	66	NR	NR	Primary	CCE, lymphadenectomy	NR
Ishii et al.	2002	female	80	NR	NR	Primary	CCE	> 21
Danikas et al.	2001	female	51	Fundus with liver extension	NR	Primary	CCE, liver biopsy	< 1; DOD
Taniai et al.	1998	female	67	NR	NR	Primary	CCE, wedge resection of the liver	> 12
Fotiadis et al.	1990	female	64	NR	7 × 5 × 2	Primary	CCE	6; DOD
Egorov et al.	1989	female	73	NR	NR	Primary	NR	NR; DOD
Tarasov et al.	1987	female	55	NR	NR	Primary	NR	NR
Asamura et al.	1986	female	72	Liver and bowel infiltration	NR	Primary	CCE wedge resection of the liver, resection of transverse colon and affected parietal peritoneum	<2; DOD
Coelho et. al.	1984	female	69	NR	6,5 × 6,2	Primary	Laparotomy with CCE	NR
Willen et al.	1982	female	91	Fundus	2 cm	Primary	NR	PMD
		male	77	NR	8 cm	Primary	CCE	NR
		male	72	NR, metastasis to liver and lung	NR	Primary	NR	NR

CT: Chemotherapy; CCE: Cholecystectomy; GB: Gallbladder; DOD: Death of disease; LMS: Leiomyosarcoma; NR: Not recorded; PMD: Post mortem diagnosed.

The diagnosis of a LMS is established in accordance with the World Health Organization classification for soft tissue tumors [3]. This tumor entity consists of cells showing distinct smooth muscle features. Macroscopically, the LMS forms a white and grey coloured fleshy mass. The microscopic pattern typically consists of intersecting, sharply marginated groups of spindle cells. Usually, the LMS is immunohistochemically positive for desmin, h-caldesmon, and SMA. Immunostainings may be focally positive for CD34, epithelial membrane antigen (EMA), keratin, and S100 [3,10].

Patients who suffered from a LMS of the GB often present with abdominal pain, fever, jaundice, and weight loss [6]. In certain cases, as the one presented here, an acute or chronic cholecystitis, accompanied by cholelithiasis led to the diagnosis of a GB tumor. Predisposing factors regarding the pathogenesis of the LMS may be gallstones and chronic inflammation of the GB [6]. Our review revealed that the majority [13,24] of patients were referred to the hospital suffering from gallstones with an acute or chronic inflammation of the GB [1,2,7,11,12,14,15,17,19,20,23].

As a diagnostic approach, an ultrasound examination, a CT scan, as well as a PET-CT scan are recommended. The LMS may occur as a polypoid mass protruding into the lumen with an irregularly thickened wall. Nevertheless, the lack of specific radiological features makes the differentiation from an adenocarcinoma challenging [6].

Similar radiological, histological and immunohistochemical features make the adenocarcinoma, the rhabdomyosarcoma, the liposarcoma, the Kaposi sarcoma, and the angiosarcoma an important differential diagnosis to consider [12,13].

The therapy depends on the tumor extension. In a non-metastatic stage, the cholecystectomy combined with a wedge resection of the surrounding liver tissue, as well as a lymphadenectomy of the hepatoduodenal ligament seems to be a sufficient surgical approach [6,9,10,15,19,25]. Contrarily, Guo et al. and Perez-Montiel et al. treated a LMS of the GB by performing a solely cholecystectomy. Their patients survival rate was about 2 years [8,14]. Our review revealed that an extensive surgery was performed in cases of a local tumor invasion [1,10,13]. There is not sufficient evidence in the literature regarding the effectiveness of adjuvant chemo- or radiation therapy. However, some authors reported that chemotherapy with doxorubicin, mitomycin C may improve the long term survival following surgery [1,17,26].

The LMS of the GB has a very poor prognosis, particularly in a metastatic stage [1,17]. By removing the LMS in an early tumor stage, a long term survival for several years, as shown in our case report, has been described [8,14].

## 4 Conclusion

Particularly when diagnosing a tumor of the GB in elderly women, a leiomyosarcoma should be taken under consideration.

In a non-metastatic stage, the cholecystectomy with a wedge resection of the surrounding liver tissue, as well as a lymphadenectomy of the hepatoduodenal ligament is described as a sufficient surgical approach.

## Funding

Publication fee will be paid HELIOS Forschungsförderung of the Helios concern.

## Ethical approval

No ethical approval necessary.

## Consent

I have obtained written consent for publication of this case report from the patient and I can provide this should the Editor ask to see it.

## Author contribution

Dr. med. Christoph Paasch (corresponding author): Contribution to the paper: author, data collection, data analysis and interpretation, writing the paper.

Dr. med. Muharrem Salak (co-author): Contribution to the paper: surgical treatment of the patient.

PD Dr. med. Thomas Mairinger (co-author): Contribution to the paper: data analysis.

PD Dr. med. Franz Theissig (co-author): Contribution to the paper: Histopathological examination, interpretation of the histological pictures, immunohistochemically examination.

## Registration of research studies

The case report at hand is not a first-in-man case report of a novel technology or surgical technique, therefore a registration of these case reports according to Declaration of Helsinki 2013 is not required.

## Guarantor

Dr. med. Christoph Paasch.

Helios Klinikum Berlin-Buch.

Department of General, Visceral and Cancer Surgery, Schwanebecker Chaussee 50, Berlin.

## Provenance and peer review

Not commissioned, externally peer-reviewed.

## Declaration of Competing Interest

None.
